# New Insights into the Morphological Diversity of *Saprolegnia parasitica* (Oomycota) Strains under In Vitro Culture Conditions

**DOI:** 10.3390/jof9100982

**Published:** 2023-09-29

**Authors:** Noémi Erdei, Tímea Hardy, Viktória Verebélyi, András Weiperth, Ferenc Baska, Edit Eszterbauer

**Affiliations:** 1 HUN-REN Veterinary Medical Research Institute, 1143 Budapest, Hungary; erdei.noemi@vmri.hun-ren.hu (N.E.); hardy.timea@vmri.hun-ren.hu (T.H.);; 2 Department of Freshwater Fish Ecology, Institute of Aquaculture and Environmental Safety, Hungarian University of Agriculture and Life Sciences, 2100 Gödöllő, Hungary; 3 Department of Exotic Animal and Wildlife Medicine, University of Veterinary Medicine Budapest, 1078 Budapest, Hungary

**Keywords:** water mold, fish parasites, saprolegniosis, *S. parasitica* morphotypes, genotypes, RPB2, SHMT gene

## Abstract

*Saprolegnia parasitica* Coker, 1923 is a primary fish pathogen and one of the most common water molds in freshwater ecosystems. In our study, nineteen strains of *S. parasitica* were isolated, identified, and characterized using morphological and genetic markers. On the basis of the abundance of zoosporangia, gemmae, the formation of gemma chains, and the induction of zoospore release, three morphotypes were differentiated. A species-level molecular identification of isolates was performed using the ITS 1 and 2 regions. A total of six genotypes were distinguished based on partial DNA sequences of the genes RNA polymerase II subunit B (RPB2) and serine hydroxymethyltransferase (SHMT). In five settings of in vitro culture conditions differing in the mineral content and the temperature of water and in the presence of a host or bait, we found that the addition of fish skin extract boosted the formation of asexual reproductive and persistent vegetative structures in cultures, whereas an unfavorable environment did not support the formation of these structures in vitro.

## 1. Introduction

The water mold *Saprolegnia* species belong to the fungal-like organisms, the oomycetes (Oomycota), and an SSU-rRNA-based phylogeny revealed they are more related to brown algae and diatoms than to fungi [1]. Although oomycetes are mainly saprotrophic, many of them infect a broad range of animals and plants [2]. Studies on *Saprolegnia* have been of increasing interest lately, especially those focused on the identification and characterization of pathogenic species such as *Saprolegnia parasitica* Coker, 1923. The identification of *S. parasitica*, however, can be complicated and troublesome, mainly because species description is largely based on the sexual reproductive structures, such as oogonia, oospores, and antheridia, and many isolates, especially those from animals, often fail to produce these sexual structures in vitro [3,4,5,6]. Thus, it is not surprising that morphology-based identification has taken a back seat to molecular methods. However, notable achievements were reached in this matter when researchers managed to induce the formation of asexual and sexual reproductive structures under different laboratory conditions [7,8,9]. A common view is that the sexual reproductive structures (i.e., oogonia and antheridia) are formed under unfavorable conditions in vitro [10,11,12]. On the other hand, depending on the environmental conditions, oogonia and antheridia may be rare or even absent in in vitro cultures, as others have found [7,8]. Sometimes cultures tend to yield abortive oospores in nutrient-poor environments, whereas normal oogonia and oospores may re-appear in the presence of a host organism when conditions become favorable [13,14].

DNA-sequence-based molecular identification has opened up new possibilities for making the *Saprolegnia* taxonomy more robust. The continuously expanding DNA sequence databases have also created new technical possibilities for species identification at a DNA level [15,16,17,18,19]. However, the public databases (such as GenBank) store several sequences with errors or even sequences of misidentified species [5,20]. Accurate species delimitation is critical for most biological disciplines. Therefore, the use of molecular operational taxonomic units (MOTUs) was aimed at solving the issue of species delimitation, although MOTUs may be insufficient for the differentiation of *Saprolegnia* strains/isolates due to the high genetic similarities among internal transcribed spacer (ITS) sequences [5,21]. DNA-level intraspecific differentiation of *S. parasitica* was performed by Ravasi et al. [22]. They developed a multilocus sequence typing (MLST) scheme that was found to be a suitable technique for the genotyping of *S. parasitica* for molecular epidemiology purposes and for the analysis of population structure. They examined 77 isolates from various geographic areas in Switzerland and differentiated 14 diploid sequence types (DSTs), of which one was found to be responsible for the majority of saprolegniosis outbreaks in the studied area.

In the present study, our goal was to define an efficient and reliable method for inducing the formation of reproductive and persistent vegetative (i.e., gemmae and gemma chains) structures of *S. parasitica* under in vitro conditions. Using the *S. parasitica* isolates collected, identified, and characterized in the present study, we aimed to determine how nutrition, temperature, and the presence of a potential host influenced the development of different isolates. Furthermore, we intended to study whether changes in the quantity and morphology of gemmae, zoosporangia, and zoospores/cysts correlated with the genetic diversity of the examined isolates.

## 2. Materials and Methods

### 2.1. Isolation of Water Mold

Water molds were isolated from water samples, infected adult fish, hatched larvae, or fish eggs. Most of the water mold samples originated from fish farms: from the trout hatchery in Lillafüred, Hungary (48°7′19.31″ N, 20°34′50.30″ E), and from carp hatcheries located in Dinnyés (47°10′58.98″ N, 18°32′22.23″ E), Varsád (46°31′51.27″ N, 18°30′36.43″ E), and Akasztó (46°44′4.23″ N, 19°10′57.75″ E), Hungary. Furthermore, some of the samples were collected at the aquaculture facility of the Institute of Aquaculture and Environmental Safety, Hungarian University of Agriculture and Life Sciences, Gödöllő, Hungary (Table 1).

For water samples, sterilized hemp seeds were incubated in 500 mL samples for 3 days at room temperature (RT; +20–21 °C) until water mold hyphae became visible on the surfaces of seeds, as described by Eszterbauer et al. [23]. The inoculated hemp seeds and every other sample type (i.e., eggs, hatched larvae, or pieces of affected fins) were placed onto glucose–yeast extract agar (GY + P + S) containing 10 g/L glucose, 2.5 g/L yeast extract, 15 g/L agar, 500 mg/L penicillin G sodium salt (P), and 500 mg/L streptomycin sulfate (S). For some cases, samples were taken with sterile cotton swabs from host skin, and swab heads were placed on GY + P + S agar. Plates were incubated at RT for about 2 days until *Saprolegnia* growth was clearly visible. Subsequently, pure *Saprolegnia* cultures were prepared by sub-culturing according to the protocol outlined by Eszterbauer et al. [24]. All isolates were maintained on GY + P + S agar and stored in a fridge at +6–8 °C. These cultures supplied the source of the subsequent in vitro examinations.

### 2.2. Molecular Methods

Water mold hyphae were sampled from the in vitro cultures in GY + P + S agar (approx. 10–15 mg wet weight) and collected in 200 µL of double-distilled water (ddH_2_O). For DNA extraction, the Quick-DNA Fungal/Bacterial Miniprep Kit (Zymo Research, Tustin, CA, USA) was used, following the manufacturer’s manual. The hyphae were homogenized in a BashingBead tube (containing 750 µL of BashingBead buffer) using TissueLyser LT (Qiagen, The Netherlands) at 50 Hz for 2 × 5 min. The concentration of the extracted gDNA was quantified using a NanoDrop 2000 spectrophotometer (Thermo Fisher Scientific, Waltham, MA, USA). Then, samples were stored at −20 °C until subsequent use. For species identification, the internal transcribed spacer (ITS) regions (an approximately 710 bp PCR product included a short fragment of 18S ribosomal rDNA, ITS 1, 5.8S rDNA, ITS 2, and a fragment of 28S rDNA) were amplified using a primer pair that is universal for fungi and fungal-like organisms (forward primer, ITS-1: 5′-TCC GTA GGT GAA CCT GCGG-3′, reverse primer, ITS-4: 5′-TCC TCC GCT TAT TGA TAT GC-3′) as described by White et al. [25], with a modified PCR protocol. Thermal cycling was performed in a Labcycler Basic (SensoQuest, Göttingen, Germany) with the following conditions: initial denaturation (at 94 °C for 5 min); followed by 6 cycles of denaturation at 94 °C for 30 s, annealing at 55 °C for 30 s, and elongation at 72 °C for 60 s; followed by 34 cycles at 94 °C for 30 s, 52 °C for 30 s, and 72 °C for 60 s; with a final elongation step at 72 °C for 10 min. PCR amplifications were performed in 25 μL containing 1× Taq buffer with KCl (Thermo Fisher Scientific, Waltham, MA, USA), 250 nM of forward and reverse primers (IDT, Leuven, Belgium), 10 mM dNTPs (Merck/Sigma-Aldrich, Darmstadt, Germany), 1.5 mM MgCl_2_ (Thermo Fisher Scientific, Waltham, MA, USA), 1.25 U recombinant Taq DNA polymerase (Thermo Fisher Scientific, Waltham, MA, USA), and approximately 10–30 ng of template gDNA.

For the genetic differentiation of *S. parasitica* isolates, seven housekeeping genes, alanyl-tRNA synthetase (ALTS1), cytochrome c oxidase subunit 1 (COX1), glutaminase (GLUT), NADH dehydrogenase subunit 1 (NAD1), RNA polymerase II subunit B (RPB2), serine hydroxymethyltransferase (SHMT), and Beta tubulin (TUBB), were tested. They were previously used for genotyping by Ravasi et al. [22]. The COX1, GLUT, NADH, and TUBB sequences were almost identical in our isolates. In the case of ALTS1, we could not obtain clear DNA sequences for all isolates, even after multiple trials, probably due to secondary conformation issues. The examined SHMT gene fragment possessed six SNPs. RPB2 performed the best, and the number of SNPs was over ten in the sample dataset of six. Therefore RPB2 and SHMT were chosen for the genetic differentiation of all examined isolates (Dataset S1).

A partial, approximately 650 bp DNA fragment of the RPB2 gene was amplified using the forward primer SAP-RPB2f: 5′-CGA CCG CGA TCA CTA TGG-3′ and the reverse primer SAP-RPB2r: 5′–CGA CAC TTC GGC GTC AAT GT–3′ according to Ravasi et al. [22], with a slightly modified protocol. The PCR conditions comprised an initial denaturation (at 95 °C for 5 min); followed by 40 cycles of denaturation (at 95 °C for 30 s), annealing (at 54 °C for 30 s), and elongation (at 72 °C for 60 s); with a final elongation step at 72 °C for 7 min. For the amplification of a 486 bp fragment of SHMT, the primer pair SHMTf: 5′–CAA GCC GCT CAA GGA GAC–3′ and SHMTr: 5′–CGT GTC GTA GTC GAT CAA GC–3′ was used with the same PCR conditions as for the RPB2 gene (except that the annealing temperature was 58 °C). PCR amplifications were carried out using the same device and reagents as described above. All PCR products were purified using a MEGAquick-spin Plus Total Fragment DNA Purification Kit (Intron Biotechnology, Seongnam, Gyeonggi, Republic of Korea) according to the manufacturer’s manual. Sanger sequencing was performed using a BigDye Terminator v3.1 Cycle Sequencing Kit (Life Technologies, Carlsbad, CA, USA) and was detected using an Applied Biosystems Genetic Analyzer 3500 (Thermo Fisher Scientific, Waltham, MA, USA).

The molecular species identification was based on an ITS DNA sequence similarity search using NCBI Megablast. A concatenated alignment was created using consensus RPB2 and SHMT DNA sequences (and relevant *S. parasitica* sequences from NCBI Genbank) obtained with the MAFFT alignment tool in Geneious Prime v2019.2.1 (Dataset S1 and Table S3). The best evolutionary model was predicted using ModelTest-NG v0.1.5 and was shown to be GTR + I + G4 for RPB2 and GTR + I for SHMT. The tree inference was conducted using RAxML-NG v1.1.0. The robustness of the tree was determined with a non-parametric, transfer bootstrap expectation (TBE) calculation using 1000 repeats. *S. ferax* isolate SAP213B was chosen as an outgroup. The phylogenetic tree was visualized using MEGA 7 and edited using Inkscape v1.3. The threshold bootstrap value for distinguishing genotypes was 75%. In addition, the genotype-related pattern of single-nucleotide polymorphisms (SNPs) was determined.

### 2.3. In Vitro Examinations

To optimize the in vitro-induced gemma and zoosporangium formation of *S. parasitica*, six isolates (SAP198, SAP191, SAP203T, SAP236, SAP134, and SAP204) were selected based on their diverse geographic origins and sample sources (i.e., water, egg, or fish samples). Five trials with different settings were carried out at two temperatures (RT at +20–21 °C vs. a chilled fridge temperature at +6–8 °C). Each setting was applied in three replicates per isolate. The components of the media were used in an SDW setup (sterile distilled water (SDW) only); an SDW + HS setup (SDW with hemp seeds added); an SDW + FS setup (SDW with a fish skin preparation added (i.e., carp skin about 5 × 5 mm in size)); an STW + HS setup (sterile, de-chlorinated tap water (STW; Table S1) with hemp seed); and an STW + FS setup (STW with fish skin preparation). All wells contained antibiotics (500 mg/L penicillin G sodium salt and 500 mg/L streptomycin sulfate).

From the cultures of all *S. parasitica* isolates examined, agar plugs 4 mm in diameter were taken with a sterile biopsy punch, and the plugs (one plug per well) were placed into 24-well plates in 1.5 mL of SDW or STW (based on the trial; see above). They were incubated for 2 weeks, and a piece of a hyphal branch from each well was examined in a squash preparation under a light microscope (Zeiss AxioStar Plus, Zeiss, Oberkochen, Germany) every three days. In the course of the microscopic examinations, we often used a 1% methylene blue aqueous solution to stain the vegetative and reproductive structures of water molds. Photomicrographs were taken using an AxioCam ERc5s microscope camera system (Zeiss, Oberkochen, Germany). Measurements of structures (i.e., sporangia, hyphae, and immature oogonia) were taken, but we found that these values varied considerably within isolates, depending on the culture conditions, the age of the culture, or even between replicates. Due to the large variation in the measurements of structures within isolates, these data could not be used for isolate characterization. Zoospore cysts were measured in the STW + FS condition at RT.

On the basis of the outcome of the trials, the “best-performed” setting was selected for comparing the in vitro growth parameters of all nineteen *S. parasitica* isolates at hand. Examinations were performed in 1.5 mL of STW containing a fish skin preparation in 24-well plates and incubated at RT for 2 weeks, with regular checks, similar to the above trials. Three replicates were applied per isolate. Besides the shapes of persistent vegetative structures (i.e., gemma and gemma chains), the relative quantity of gemmae and zoosporangia was scored in squash preparations (++++: an extremely high amount of zoosporangia covered the field of view in a light microscope (not countable); +++: a high amount of zoosporangia (at least half of the microscope field was covered); ++: a high but countable number of zoosporangia or gemmae per field/a few gemma chains per field; +: a few zoosporangia or gemmae per field/a couple of gemma chains per field; (+) gemma chains occasionally occurred; -: zoosporangia, gemmae, or gemma chains absent). In addition, the number of zoosporangia containing primary cysts was also estimated: +++: numerous zoosporangia per field; ++: a few zoosporangia per field; +: a couple of zoosporangia were found in the entire preparation; -: structure absent.

The sporulation of *S. parasitica* isolates was achieved using the protocol described by Diéguez-Uribeondo et al. [26] with modifications and after optimization. Briefly, mycelial plugs (4 mm in diameter) of *S. parasitica* were made with a biopsy punch, placed and incubated in 1.5 mL of GY + P + S liquid media (containing 10 g/L glucose, 2.5 g/L yeast extract, 500 µg/mL P, and 500 µg/mL S) in 24-well plates at about 21 °C for 3 days. The sporulation was induced by washing the mycelia with sterile-filtered distilled water (SDW) three times, then incubating in sterile-filtered, chlorine-free, tap water (STW) at 21 °C for 3 days. On day 3, mycelial plugs were washed again three times with SDW and incubated in STW for 4.5 h to induce zoospore release (RT setup). In parallel, samples were incubated in a fridge (+6–8 °C) overnight after washing. Then, the experiment proceeded the same way as for the RT setup. The quantity of zoospores and cysts was estimated by counting in a Bürker chamber (++++: >10^5^ zoospores/mL; +++: 10^4^–10^5^ zoospores/mL; ++: 10^3^–10^4^ zoospores/mL; +: <10^3^ zoospores/mL; -: no zoospores found).

## 3. Results

A total of nineteen *S. parasitica* isolates were collected, and BLAST searches based on their ITS DNA sequences confirmed that they were indeed of the species *S. parasitica*.

### 3.1. Alterations in the Morphology of Zoosporangia and Gemmae

**Figure 1:**
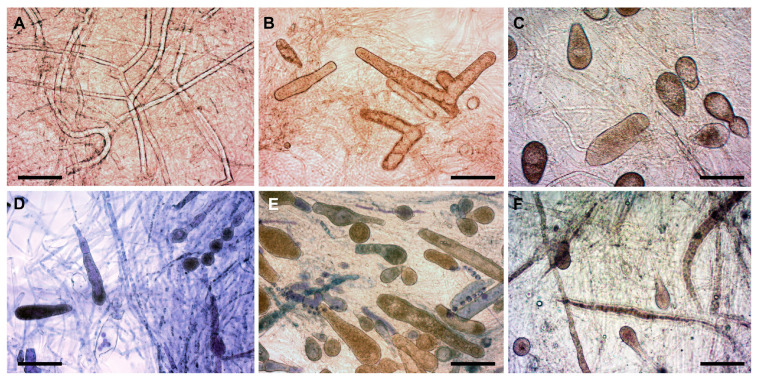
Morphological variability of *S. parasitica* (isolate SAP134) under various in vitro conditions. (**A**) SDW at RT (hyphae only); (**B**) SDW + HS at RT (zoosporangia and gemmae); (**C**) SDW + FS at RT (zoosporangia and gemmae); (**D**) STW + HS at RT (zoosporangia with or without primary cysts inside, gemma, and gemma chain); (**E**) STW + FS at RT (gemma and zoosporangia with or without primary cysts inside); (**F**) STW + FS at fridge temperature (+6–7 °C) (zoosporangia). Scale bar: 100 µm. Native squash preparations (except for (**D**,**E**), squash preparations stained with 1% aqueous methylene blue). SDW: sterile distilled water; STW: sterile, de-chlorinated tap water; HS: hemp seed; FS: fish skin extract; RT: room temperature (+20–21 °C).

The appearance of vegetative and asexual reproductive structures depended on the in vitro conditions for the six *S. parasitica* isolates examined (Table 2). In the five in vitro trials, the presence and amounts of gemmae, gemma chains, and zoosporangia differed remarkably. Moreover, for isolates SAP134 and SAP204, numerous zoosporangia with primary cysts were observed mainly in STW + FS at RT (Table 2). Overall, RT was preferred over the chilled temperature for the formation of gemmae and zoosporangia (Table 2 and Figure 1). Reproductive structures were not observed on the coenocytic branched hyphae when incubated in SDW at RT. When the incubation was performed in SDW with hemp seeds added, few zoosporangia and occasional gemmae were observed. If the incubation was in STW with hemp seeds added, zoosporangia and gemmae appeared in greater numbers and some gemma chains were also observed in certain isolates (e.g., SAP198, SAP134, and SAP204). The highest numbers of gemma, gemma chains, and zoosporangia were observed when the hemp seeds were replaced by the fish skin preparation (Table 2).

Some exceptions could be recognized in terms of environmental tolerance. Isolates SAP198, SAP203T, and SAP236 developed zoosporangia well at both room and fridge temperatures. Furthermore, primary zoospores/cysts remaining in sporangia were observed for isolate SAP134 (in both STW and SDW with HS and FS, even at the cold temperature), SAP198 (SDW in the presence of FS at the cold temperature), and SAP204 (in STW and with FS at both temperatures). Overall, however, a nutrition-rich, temperate environment (i.e., STW supplemented with fish skin at RT) was preferred over conditions low in nutrients, minerals, and/or temperature (Table 2). Therefore, the former in vitro environment was applied to examine the growth performance of all *S. parasitica* isolates, and we found notable differences in the numbers of asexual reproductive and persistent vegetative structures among the isolates (Table 3).

### 3.2. S. parasitica Morphotypes and Their Relations to Genotypes

**Figure 2:**
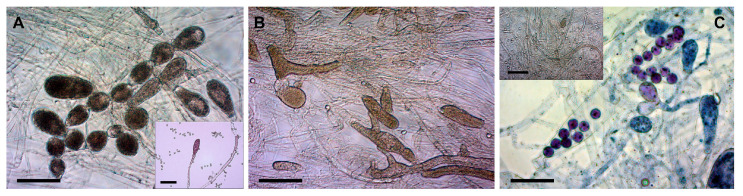
Morphological differences among the three morphotypes of *S. parasitica* (conditions: STW + FS at RT). (**A**) Long gemma chains in Morphotype 1 (SAP194); A inset: zoosporangium and cysts; (**B**) gemmae, zoosporangia, zoospores, and cysts in Morphotype 2 (SAP203T); (**C**) primary cysts in a zoosporangium in Morphotype 3 (SAP134); C inset: numerous zoospores and cysts produced after cold induction (SAP200B). Scale bar: 100 µm. Native squash preparations (except for (**C**), squash preparations stained with 1% aqueous methylene blue).

**Figure 3:**
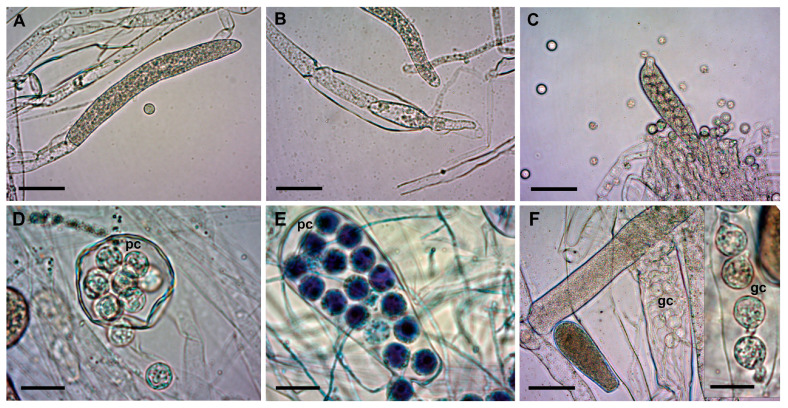
Zoosporangia of *S. parasitica* at different stages of development. (**A**) Primary, immature zoosporangium and a cyst (SAP194); (**B**) secondary zoosporangium (SAP191); (**C**) mature zoosporangium with numerous zoospores and cysts around (SAP191); (**D**) spherical zoosporangium; (**E**) clavate zoosporangium with filiform appendix in the last stage of zoospore release (SAP200B); (**F** and **F** inset): cysts germinating in zoosporangia (SAP200B). Scale bars: 50 µm (**A**–**C**,**F**) or 20 µm (**D**–**F** inset). pc: primary cysts; gc: germinating cysts. Squash preparations, native (**A**–**D**,**F** and **F** inset) or stained with 1% aqueous methylene blue (**E**).

Nineteen *S. parasitica* isolates were divided into three morphotypes according to their propensities to form gemmae, gemma chains, and zoosporangia in STW containing fish skin extract at RT and taking into account zoospore production in STW without fish skin at RT and at fridge temperature (Table 3 and Figure 2):

- Morphotype 1 (SAP194, SAP198, SAP214A, SAP191, and SAP207B) (Figure 2A): very prone to develop zoosporangia; zoospore production preferred at RT; long gemma chains of 5–7 gemmae are common.
- Morphotype 2 (SAP147B, SAP208B, SAP199, SAP203T, SAP206, SAP236, and SAP235) (Figure 2B): prone to develop zoosporangia; moderate numbers of zoospores at RT; gemma chains of 3–5 gemmae occur.
- Morphotype 3 (SAP139, SAP200B, SAP197, SAP215B, SAP134, SAP204, and SAP209A) (Figure 2C and Figure 3D–F): few zoosporangia; primary zoospores/cysts may remain and germinate in zoosporangia; gemma chains not characteristic; moderate zoospore production at RT; cold-induced zoospore production preferred.

The three morphotypes were clearly distinguishable based on the abundance of gemma chains and zoosporangia and the presence or absence of cysts remaining in zoosporangia (Figure 2). Morphotype 1 produced long gemma chains and zoosporangia in STW + FS at RT (Figure 2A). Gemmae and zoosporangia were abundant in Morphotype 2 (Figure 2B), whereas for Morphotype 3 primary cysts often remained and even germinated inside zoosporangia (Figure 2C).

The sexual reproductive structures, antheridium, and oogonium were not conclusively detected and were excluded from the analysis. Conversely, zoosporangia with remaining primary cysts inside were another feature that helped in the differentiation of morphotypes. The isolates of Morphotype 1 did not develop these structures at all, whereas in Morphotype 3 isolates, medium to high amounts of zoosporangia with primary zoospores and cysts were observed (Table 3; see measurements of cysts in Table S2). In some cases, cysts were germinating in the sporangia, showing an “aplanoid-like” zoospore emergence (Figure 3F). Nevertheless, a saprolegnoid zoospore discharge was the most common in all three morphotypes.

Although the in vitro conditions were the same (STW + FS at RT), the shapes of zoosporangia were rather diverse (Figure 3). We found filiform-, elongate-, spherical-, pyriform-, and clavate-shaped zoosporangia, sometimes even in the same culture. Similarly, gemmae also had various shapes (clavate, pyriform, and irregular), which were often catenulate and occasionally intercalary.

For Morphotype 1, zoospore production was intense (>10^4^ zoospores/mL) at RT, whereas isolates of Morphotype 2 produced a moderate number of zoospores, even after cold induction. The isolates of Morphotype 3 developed a moderate number of zoospores at RT; however, overnight cooling remarkably enhanced zoospore production (Figure 2C inset).

**Figure 4:**
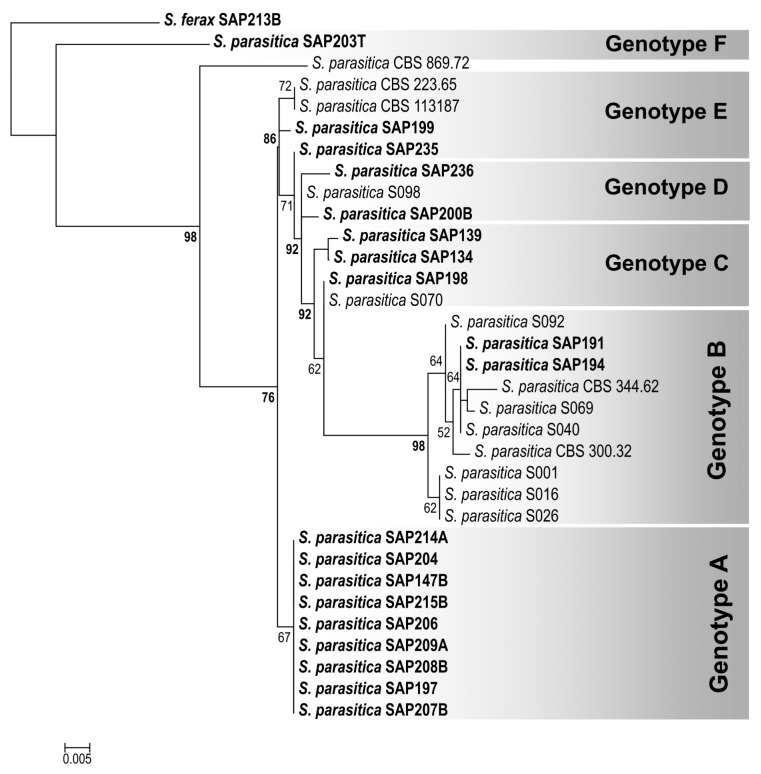
Maximum-likelihood (RAxML) tree reconstruction of *Saprolegnia parasitica* isolates examined (labeled in bold) and related DNA sequences from NCBI database, based on a 1037 bp concatenated DNA sequence alignment of RPB2 and SHMT gene fragments. *S. ferax* (SAP213B) was chosen as the outgroup. Numbers at nodes indicate the bootstrap values in percentages (shown above 50%; the bootstrap values above the genotype threshold, i.e., 75%, are labeled in bold). NCBI accession numbers are listed in Table S3.

The DNA sequence similarities of the isolates varied between 99.15% and 100% for ITS, whereas for RPB2 and SHMT, 93.65–100% and 98.56–100% identities were detected, respectively. The consensus DNA sequences of the examined *S. parasitica* isolates were submitted to the NCBI database (GenBank) under the accession numbers listed in Table 3. ITS sequences were obtained for the species-level identification of isolates, and they confirmed that all 19 isolates used for in the vitro examinations belonged to *S. parasitica*. In the 611 bp fragments of the RPB2 gene, 27 SNPs were recognized in total, whereas 7 SNPs were detected in the 416 bp fragments of SHMT (Dataset S1). Among the isolates, six *S. parasitica* genotypes (RPB2/SHMT genotypes A–F) were distinguished, supported by the concatenated maximum-likelihood phylogenetic tree reconstruction (Figure 4) and the SNP patterns of the genotypes (Table S4). RPB2/SHMT genotype B was found among the isolates of Morphotype 1, whereas genotypes E and F were only present in Morphotype 2. The isolates of genotype A, often with 100% identical RPB2 and/or SHMT DNA sequences, were evenly distributed among all three morphotypes, indicating that there is no correlation between the morphotypes and the RPB2/SHMT genotypes (Table 3).

## 4. Discussion

In the present study, a total of nineteen *S. parasitica* isolates were examined using morphological and genetic markers; their environmental preference was investigated, and morphotypes and genotypes were distinguished. Our study focused on the asexual reproductive structures (zoosporangium and zoospores/cysts), but attention was also paid to the persistent vegetative structures (gemmae and gemma chains). Sexual reproductive organs were not observed in any of the isolates or circumstances in our study. Similar results were reported by Vega-Ramírez et al. [8] when sexual reproduction was not observed in any of the 34 *S. parasitica* isolates examined or by Yuasa and Hatai [7], who found that 1 of 15 *S. parasitica* isolates produced oogonia after two weeks of incubation.

Based on data from the literature and our own experience, five culture conditions varying in mineral contents, the temperature of water, and the presence of a host or bait were tested to find the most suitable in vitro environment, in which the highest numbers of reproductive and vegetative structures developed in the isolates studied. In terms of temperature, the original condition (i.e., the condition at the time of sampling; Table 1) was often different from the one in the laboratory. Isolates from Lillafüred trout hatchery (SAP191 and SAP194) were collected from hatchery water at +9 °C, but they preferred RT over the colder temperature (+6–7 °C) under in vitro conditions. Conversely, sample SAP198 was collected at a carp farm in a much milder condition (+15 °C), but this isolate seemed to have a broader thermal tolerance, as it produced numerous zoosporangia at both temperatures (+6–7 °C and +20–21 °C). SAP134 originated in cold water (+3 °C) but mainly produced zoosporangia and gemmae at RT. However, these trends in temperature preference were only observations and were not confirmed experimentally, as the behavior of the isolates was not tested at the water temperatures measured at the time of sampling.

Previous studies concluded that the formation of reproductive structures is favored in a nutrient-poor environment under laboratory conditions [10,11,12]. Their concept is that the lack of proper nutrition is a stress factor and the reproduction of water molds is initiated as a reaction to environmental stress, which sounds reasonable. In our study, however, reproductive structures were not observed in any *S. parasitica* morphotypes when incubated in SDW at RT, even though distilled water most likely caused osmotic stress in addition to the severe deprivation of nutrition. In the detailed monograph by Johnson et al. [3], the authors highlighted that factors such as medium composition, temperature, light, and pH were associated with the physiology of reproductive cell development; moreover, the presence of particular inorganic salts favored the development of the reproductive apparatus. Referring to studies from early times in the 20th century, they stated that different combinations of carbon and nitrogen sources might alter the preferences of reproductive structures. Although sexual reproductive structures were not detected in our study, our findings show that the presence of fish skin is more effective in boosting the differentiation of asexual reproductive structures (i.e., zoosporangia) than the addition of hemp seeds or the absence of both. Under the STW + FS condition, along with the inorganic salt content of tap water, organic elements (proteins, lipids, and sugars) were present in the fish skin preparation. Potential signaling molecules (e.g., nucleosides, oligopeptides, etc.) could also be available in fish skin, which might trigger the growth or even the host recognition of *S. parasitica*, as is the case for another aquatic parasite taxon, the Myxozoa [27,28].

Among the three morphotypes distinguished in the liquid medium of STW with fish skin (STW + FS), a notable difference was found in terms of the abundance of gemmae and gemma chains. A gemma is a vegetative structure that provides—among other things—the survival of water molds under unfavorable conditions [3,29,30]. Conversely, some authors reported that a nutrient-rich condition might also induce gemma formation [3,10]. Gemmae can have various shapes and are able to form chains of different lengths, as shown in our study. We reckon that gemmae with a loose internal structure may represent a pre-differentiation stage, when they start to develop into other structures (e.g., zoosporangium). Seymour [29] found that gemmae of *S. parasitica* can develop into oogonia or zoosporangia, and his observation seems to support our theory, even if it has not been confirmed experimentally. Gemmae are known to be capable of fragmentation [30], and fragments may then spread by water flow (or in vitro in liquid media), so this is probably one of the means of propagation (in addition to spreading via zoospores). We have not studied the function of gemmae in in vitro cultures or how (if at all) they might develop further. However, the high abundance of gemmae and gemma chains under “host-like” conditions (i.e., STW + FS) suggests that these vegetative structures may be associated with the dissemination of *S. parasitica*. In vivo infection experiments may provide an answer.

Morphotype 3 was characterized by the frequent occurrence of zoospores containing primary cysts, and in many cases cyst germination was also observed, which resembled an aplanoid zoospore release. In *Saprolegnia* species, the saprolegnoid type of zoospore discharge is the most common way of releasing primary zoospores from sporangia, whereas the aplanoid type is typical of *Aplanes* spp. and is even named after this genus [31]. In addition, this process is often observed for *Achlya* spp., such as *Achlya abortispora* [32] and *Achlya spiralis* [33]. However, as reported previously, discharge may also be aplanoid or dictyoid for some *Saprolegnia* species, such as *S. ferax*, *S. diclina*, *S. parasitica*, and *S. turfosa* [3,31]. One argument is that aplanoid zoospore discharge occurs; thus, the in situ germination of zoospores may occur under suboptimal environmental conditions or in “old” *Saprolegnia* cultures [3,34]. Our theory is that besides these factors, the lack of sufficient mechanical “disturbance” in in vitro cultures may also cause this artificial, aplanoid-like discharge, and the mechanical impacts (even the one during the washing steps) may aid zoospore release; thus, zoospores are able to leave the sporangia completely. However, we observed morphotype-related differences in this respect, so mechanical impacts of various intensities may be necessary for zoospore release.

Another important difference between the identified morphotypes was the nature and intensity of the zoospore emergence. Since waterborne, actively moving secondary zoospores are mainly responsible for the spread of saprolegniosis, the identification of factors influencing sporulation is of epidemiological importance. The sporulation and zoospore release of oomycetes are well studied, even experimentally; thus, some factors that increase zoospore release have already been identified, such as nutrient deficiency or low salinity [3,20]. Various techniques have been tested to enhance zoospore release under in vitro conditions, such as incubation in pond or aquarium water, repeated washing steps, or mechanical treatment [7,26,35,36,37,38,39,40]. However, for the *S. parasitica* isolates examined, none of the protocols were found to be truly effective. In a recent study, Pavic et al. [40] reported that the physico-chemical properties of the water influenced the sporulation of *S. parasitica*. They found that the addition of humic acid to natural water samples might promote zoospore formation, although in vitro tests using artificial water have not produced the same yield. In our study, the sporulation assay described by Diéguez-Uribeondo et al. [26] was modified and optimized, another washing step was included, and the effect of cold induction was tested. Zoospore release could be enhanced by sudden temperature drops, as has been shown in the case of *Saprolegnia* sp., which caused winter saprolegniosis in channel catfish [20,41]. In the present study, we found that for Morphotype 3, the key step was the overnight cold induction, which resulted in increased zoospore release. Thus, it was one of the features that distinguished Morphotype 3 from the other two.

In a previous study by Ravasi et al. [22], an MLST procedure was described for distinguishing *S. parasitica* DSTs. However, we used the genes RPB2 and SHMT for genotyping. The aim of our study was to detect the morphotype-related genetic differences that were most able to discriminate among the isolates examined here. Therefore, we decided to choose the genetically diverse genes of the seven housekeeping genes examined, which in our case were RPB2 and SHMT. The three identified morphotypes of *S. parasitica* were composed of six RPB2/SHMT genotypes, with no correlation detected. Our study showed that a morphotype-related, genetic discrimination of the examined *S. parasitica* isolates was not possible using these coding genes. It is also likely that housekeeping genes are not suitable discriminative markers for such purposes. Thus, transcriptome sequencing and high-throughput gene expression studies could provide a means of identifying genetic markers that are more suitable for the examination of morphotypes with behavioral differences in response to in vitro environmental changes.

## 5. Conclusions

In the present study, the effects of culture nutrient and mineral content, water temperature, and the presence of a potential host on the development of asexual reproductive and persistent vegetative structures of several *S. parasitica* isolates were examined in vitro. Taking these parameters into account, we conclude that STW + FS (i.e., tap water with the addition of a fish skin preparation), as a culture medium, is a suitable environment for the examination of vegetative and asexual reproductive structures of *S. parasitica*. Under this condition, the examined *S. parasitica* isolates presented clear morphological differences in the formation of gemmae, gemma chains, and zoosporangium. In addition, notable differences in the method and amount of zoospore release could be detected between the three morphotypes distinguished. The morphotypes did not show a correlation with the RPB2/SHMT-based genotypes. Therefore, further studies will aim to identify genetic markers more suitable for the morphotype-based differentiation of *S. parasitica*. Furthermore, pathogenicity-related examinations of *S. parasitica* morphotypes are also planned.

## Figures and Tables

**Table 1:** *Saprolegnia parasitica* isolates collected in the present study.

Sample ID	Geographic Location (in Hungary)	Sample Type	Host Species	Water Temperature * (°C)
SAP134	Gödöllő	water	-	3
SAP139	Gödöllő	water	-	3
SAP147B	Akasztó	egg and larvae	common carp (*Cyprinus carpio*)	22
SAP191	Lillafüred	eyed egg	rainbow trout (*Oncorhynchus mykiss*)	9
SAP194	Lillafüred	water	-	9
SAP197	Varsád	skin scrapings	common carp (adult)	15
SAP198	Varsád	skin scrapings	common carp (adult)	15
SAP199	Varsád	skin scrapings	common carp (adult)	15
SAP200B	Varsád	dorsal fin	common carp (adult)	15
SAP203T	Dinnyés	skin	common carp (larvae)	21
SAP204	Dinnyés	skin	common carp (larvae)	22
SAP206	Dinnyés	skin scrapings	common carp (adult)	22
SAP207B	Dinnyés	egg	common carp	22
SAP208B	Dinnyés	skin	common carp (larvae)	22
SAP209A	Dinnyés	water	-	22
SAP214A	Dinnyés	water	-	22
SAP215B	Dinnyés	egg	European catfish (*Silurus glanis*)	22
SAP235	Gödöllő **	caudal fin	three-spined stickleback (*Gasterosteus aculeatus*) (adult)	22
SAP236	Gödöllő **	skin scrapings	three-spined stickleback (adult)	22

* Water temperature at the time of sampling. ** The fish specimens were originally captured in natural water, Gombás brook, near Vác, Hungary, but were then kept at the aquaculture facility of the Institute of Aquaculture and Environmental Safety, Hungarian University of Agriculture and Life Sciences, Gödöllő, Hungary.

**Table 2:** The outcomes of in vitro trial series examining the morphological alterations among some selected *Saprolegnia parasitica* isolates. The highest numbers of vegetative and asexual reproductive structures developed in tap water (STW) in the presence of fish skin (FS) at RT (labeled in bold).

Isolates	Vegetative Structures	Trials at RT (+20–21 °C)					Trials at +6–7 °C				
		SDW	SDW + HS	SDW + FS	STW + HS	STW + FS	SDW	SDW + HS	SDW + FS	STW + HS	STW + FS
SAP198	zoosporangium	-	+	+++	+	**++++**	+	++	++	++	+++
	gemma chain *	-	-	-	+	**++**	-	+	-	-	+
	cysts in sporangium	-	-	-	-	**-**	-	-	+	-	-
SAP191	zoosporangium	-	-	+	+	**++++**	-	-	+	+	-
	gemma chain	-	-	-	-	**+**	**-**	-	-	-	-
	cysts in sporangium	-	-	-	-	**-**	-	-	-	-	-
SAP203T	zoosporangium	-	-	++	+	**+++**	-	-	++	++	+++
	gemma chain	-	-	-	-	**+**	-	-	-	-	-
	cysts in sporangium	-	-	-	-	**-**	-	-	-	-	-
SAP236	zoosporangium	-	+	++	+	**+++**	-	++	++	++	+
	gemma chain	-	-	-	-	**-**	-	-	-	-	-
	cysts in sporangium	-	-	-	-	**-**	-	-	-	-	-
SAP134	zoosporangium	-	+	++	++	**++**	-	+	+	++	+
	gemma chain	-	-	-	+	**-**	-	-	-	-	-
	cysts in sporangium	-	+	-	+	**+++**	-	-	-	+	-
SAP204	zoosporangium	+	+	+++	++	**++**	-	+	+	+	++
	gemma chain	-	-	+	+	**+**	-	-	-	-	-
	cysts in sporangium	-	-	-	-	**+**	-	-	+	-	+

SDW: sterile distilled water; STW: sterile tap water; HS: hemp seed; FS: fish skin; * gemmae were also examined in all in vitro settings, but the number of gemma chains was only estimated.

**Table 3:** The main characteristics of the three morphotypes of *S. parasitica* distinguished in the present study and their relations to the genotypes. Gemma chain and zoosporangium productions were compared in STW containing fish skin extract (FS) at RT. Zoospore release was examined in two conditions: at RT and after overnight incubation at a cold temperature (+6–7 °C), both in STW without FS. NCBI accession numbers of ITS regions and RPB2 and SHMT gene sequences are listed.

Morphotypes	Isolate ID	Sample Type	RPB2/SHMT Genotype	Gemma Chain	Zoosporangium	Cysts in Sporangium	Zoospore Released (at RT)	Zoospore Released (Cold-Induced)	NCBI Accession Nos.: ITS/RPB2/SHMT
Morphotype 1	SAP194	W	B	++	++++	-	++++	++++	OQ236394/OQ270775/id. SAP191
	SAP198	F	C	++	++++	-	++++	++++	OQ236398/OQ270773/OR503102
	SAP214A	W	A	++	++++	-	+++	+	OQ236386/OQ270766/id. SAP200B
	SAP191	E	B	+	++++	-	+++	++	OQ236393/OQ270774/OR503101
	SAP207B	E	A	+	+++	-	++++	+	OQ236389/OQ270764/id. SAP200B
Morphotype 2	SAP147B	E	A	++	+++	-	++	+	OQ236392/OQ270760/id. SAP200B
	SAP208B	F	A	+	+++	-	++	+	OQ236387/OQ270767/id. SAP200B
	SAP199	F	E	+	+++	-	+	++	OQ236399/OQ270771/id. SAP236
	SAP203T	F	F	+	+++	-	+	+	OQ236391/OQ270776/OR503104
	SAP206	F	A	+	+++	+	+	++	OQ236384/OQ270768/id. SAP200B
	SAP236	F	D	-	+++	-	+	+	OQ236397/OQ270772/OR503105
	SAP235	F	E	+	+++	-	++	+	OQ236401/OQ270770/id. SAP236
Morphotype 3	SAP139	W	C	+	+++	++	+	++++	OQ236390/OQ270759/id. SAP191
	SAP200B	F	D	(+)	++	+++	++	++	OQ236396/OQ270769/OR503103
	SAP197	F	A	(+)	++	+	++	+++	OQ236395/OQ270763/id. SAP200B
	SAP215B	E	A	(+)	++	+	+	++	OQ236385/OQ270765/id. SAP200B
	SAP134	W	C	-	++	+++	+	+++	OQ236383/OQ270758/OR503100
	SAP204	F	A	+	++	+	+	+++	OQ236400/OQ270761/id. SAP236
	SAP209A	W	A	-	++	-	-	-	OQ236388/OQ270762/id. SAP200B

W: water sample; F: fish; E: fish eggs; STW: sterile-filtered tap water; id. identical SHMT DNA sequence.

## Data Availability

All data analyzed in this study are cited and are summarized in the manuscript.

## Supplementary Materials

The following supporting information can be downloaded at https://www.mdpi.com/article/10.3390/jof9100982/s1. Table S1. Physico-chemical properties of drinking (tap) water used for in vitro assays. Table S2. Basic morphometrics of primary and secondary cysts (diameter in µm (n = 30)). Table S3. NCBI accession numbers of *S. parasitica* DNA sequences used for maximum-likelihood (RAxML) phylogenetic tree reconstruction. Table S4. SNPs detected in RPB2 and SHMT gene fragments on the basis of a 1037 bp concatenated alignment. Dataset S1. Concatenated DNA sequence alignment of RPB2 and SHMT gene fragments of *Saprolegnia parasitica* isolates examined and related DNA sequences from NCBI database (labeled with the identifier of isolates).
