# Supplementary material for: New Insights into the Morphological Diversity of Saprolegnia parasitica (Oomycota) Strains under In Vitro Culture Conditions
Source: J Fungi (Basel). 2023 Sep 29;9(10):982. doi: 10.3390/jof9100982 (PMC10607735; doi:10.3390/jof9100982)
Supplement: Supplementary file 1 [file jof-09-00982-s001.zip › jof-2555088-supplementary/TableS1-S4_Erdei-et-al2023.pdf]

Supplementary Materials

Erdei et al. Journal of Fungi

Table S1. Physico-chemical properties of drinking (tap) water used for *in vitro* assays

Components	Amount	Unit of measurements
Chloride (Cl^-)	20	mg/l
Iron (Fe)	7	µg/l
Manganese (Mn)	1	µg/l
Nitrate (NO^{3-})	7	mg/l
Nitrite (NO^{2-})	<0.03	mg/l
Ammonia (NH_3)	<0.04	mg/l
Total hardness	124	mg/l CaO
Conductivity	427	µS/cm
pH	7.6	

Table S2. Basic morphometrics of primary and secondary cysts; diameter in µm (n=30).

	Mean	±SD	Min–Max
primary cysts remaining in sporangia	16.25	±1.87	13.18–21.98
secondary cysts	11.26	±0.62	9.49–12.34

Table S3. NBCI accession numbers of *Saprolegnia parasitica* strains used for maximum likelihood (RAxML) phylogenetic tree reconstruction. RPB2: RNA polymerase II subunit B; SHMT: serine hydroxymethyltransferase.

Isolate	RPB2	SHMT	Reference
SAP134	OQ270758	OR503100	present study
SAP139	OQ270759	OR503101	present study
SAP147B	OQ270760	OR503103	present study
SAP191	OQ270774	OR503101	present study
SAP194	OQ270775	OR503101	present study
SAP197	OQ270763	OR503103	present study
SAP198	OQ270773	OR503102	present study
SAP199	OQ270771	OR503105	present study
SAP200B	OQ270769	OR503103	present study
SAP203T	OQ270776	OR503104	present study
SAP204	OQ270761	OR503105	present study
SAP206	OQ270768	OR503103	present study
SAP207B	OQ270764	OR503103	present study
SAP208B	OQ270767	OR503103	present study
SAP209A	OQ270762	OR503103	present study
SAP214A	OQ270766	OR503103	present study
SAP215B	OQ270765	OR503103	present study
SAP235	OQ270770	OR503103	present study
SAP236	OQ270772	OR503105	present study
CBS 223.65	MH048487	MH048500	Ravasi et al. (2018)
CBS 300.32	MH048494	MH048508	Ravasi et al. (2018)
CBS 344.62	MH048488	MH048501	Ravasi et al. (2018)
CBS 869.72	MH048495	MH048509	Ravasi et al. (2018)
CBS 113187	MH048499	MH048513	Ravasi et al. (2018)
S001	MH048489	MH048502	Ravasi et al. (2018)
S016	MH048490	MH048503	Ravasi et al. (2018)
S026	MH048491	MH048504	Ravasi et al. (2018)
S040	MH048493	MH048506	Ravasi et al. (2018)
S069	MH048496	MH048510	Ravasi et al. (2018)
S070	MH048492	MH048505	Ravasi et al. (2018)
S092	MH048497	MH048511	Ravasi et al. (2018)
S098	MH048498	MH048512	Ravasi et al. (2018)

Table S4. SNPs detected in RPB2 and SHMT gene fragments on the basis of a 1037-bp concatenated alignment. RPB2: RNA polymerase II subunit B; SHMT: serine hydroxymethyltransferase.

alignment	RPB2 nt positions																											SHMT nt positions						
	#7	#25	#31	#34	#70	#76	#85	#115	#145	#148	#151	#184	#208	#280	#292	#295	#391	#406	#451	#517	#529	#532	#535	#568	#577	#580	#583	#653	#749	#791	#839	#854	#986	#995
Genotype A	A	C	C	T	A	C	A	C	T	G	C	G	G	G	G	C	T	C	T	A	T	C	C	A	C	C	G	G	C	A	C	G	G	T
Genotype B	G	G	G	C	G	T	G	T	C	A	G	B	C	C	R	G	C	Y	Y	R	C	T	G	G	Y	G	A	K	Y	G	Y	S	G	Y
Genotype C	R	G	C	T	A	C	A	C	C	G	C	G	G	G	G	S	T	C	T	A	Y	Y	C	G	T	G	A	G	Y	G	T	G	G	T
Genotype D	A	S	C	T	A	C	A	C	Y	G	C	G	G	G	R	C	T	C	T	A	T	C	C	G	Y	G	A	K	C	R	C	S	K	Y
Genotype E	A	G	C	T	A	C	A	C	T	G	C	G	G	G	R	C	T	C	T	A	Y	Y	C	G	Y	S	G	K	C	G	C	S	K	C
Genotype F	G	G	C	C	G	C	G	C	C	G	C	G	C	T	G	C	C	G	C	G	T	C	C	G	C	C	G	T	T	G	T	G	T	T
